# Research trends of the neuroimaging in aphasia: A bibliometric analysis and visualization analysis from 2004 to 2021

**DOI:** 10.3389/fnhum.2022.945160

**Published:** 2022-07-15

**Authors:** Jiaqin Huang, Yun Cao, Danli Zhang, Xiaojing Lei, Jingling Chang

**Affiliations:** Department of Neurology, Dongzhimen Hospital, Beijing University of Chinese Medicine, Beijing, China

**Keywords:** aphasia, neuroimaging, bibliometrics, visualization analysis, hotspots, frontiers

## Abstract

**Objectives:**

To review the current research status of the neuroimaging of aphasia, and reveal the hotspots and frontiers of research in this field.

**Methods:**

We searched articles related to the neuroimaging research on aphasia since Web of Science (WOS) database construction and extracted the data. CiteSpace and VOSviewer were used for the country/institution analysis, journal analysis, discipline analysis, burst keyword analysis and cited-reference cluster analysis.

**Results:**

Of the studies retrieved from WOS, 2922 studies that related to the neuroimaging of aphasia were screened and finally included 2799 articles for research. The United States of America and University of California San Francisco were the main countries and institutions in this field. Brain had the highest impact factor in both published and cited journals. Through the discipline and topic analysis of this field, the most common category was Neurosciences and Neurology. The keyword with the strongest citation strength was “functional connectivity,” and the recent burst keywords were “functional connectivity” and “network.” The co-citation network showed seven clusters greater than 100. Among the top 5 clusters, the most recently formed cluster, Cluster #2 (progressive supranuclear palsy), had an average year of 2017. The literature in the top 5 clusters mainly focused on 3 aspects, specifically, the discovery of language processing models, injury and recovery mechanisms of post-stroke aphasia (PSA), and diagnosis of primary progressive aphasia (PPA) variants.

**Conclusion:**

The results of this bibliometric study revealed the following three research hotspots in the neuroimaging of aphasia: clarifying the connotation of the most recognized language processing model, the dual-stream model, exploring the injury mechanism based on the dual-stream model and the recovery mechanism involving the left and right hemispheres of PSA, and determining the diagnostic criteria for PPA variants. A major research trend is to combine new neuroimaging technology, such as PET tracer technology, to realize the visual presentation of disease-specific proteins to improve the pathological diagnostic criteria of PPA variants. Accordingly, a visualized analysis of literature that uses CiteSpace provides a more rapid, repeatable and flexible method, which is more conducive to capturing research hotspots and emerging trends.

## Introduction

Aphasia is the impaired or loss of language ability usually caused by damage to the dominant brain hemisphere (Yu et al., 2017). Aphasia refers to the impairment of language ability due to various reasons [e.g., post-stroke aphasia (PSA), primary progressive aphasia (PPA), etc.] after the acquisition of language ability, including the expression and/or comprehension barriers in spoken and written language (Crosson et al., 2019), which has a great negative impact on the quality of life (Lam and Wodchis, 2010), and its severity can be used to predict functional recovery after stroke (Koleck et al., 2017). Therefore, it is of certain significance to find effective therapies for aphasia. However, the therapeutic efficacy for aphasia is not satisfactory due to the unknown mechanism. Thus, it is urgent to study on the mechanisms of pathogenesis and recovery in aphasia.

Language is human-specific. Because human language has some certain and unique characteristics that animals do not have, there is currently a lack of suitable animal models, which has become a bottleneck in the exploration of the pathogenesis of PSA. However, in recent years, based on the characteristics and advantages of technology, more researchers have focused on neuroimaging technology to carry out research on aphasia. As a non-invasive diagnosis and evaluation technology for neurological diseases, neuroimaging technology, such as structural magnetic resonance imaging (MRI), functional magnetic resonance imaging (fMRI), diffusion tensor imaging/diffusion tensor beam imaging (DTI/DTT), electroencephalogram (EEG) and event-related potentials (ERP), can reflect the changes of the brain network in the production, injury and recovery of language functions from the perspectives of both structure and function (Shuster, 2018). Neuroimaging can be used to unravel the neurobiological bases of behavioral improvement by mapping large-scale changes in neural activity and functional and structural connectivity. Furthermore, studies on the pathogenesis and recovery of aphasia based on neuroimaging might help to refine existing models of language organization and reorganization.

However, there is currently no bibliometric analysis of global neuroimaging research on aphasia. Therefore, it is necessary to understand the current situation of the neuroimaging research on aphasia from a macro perspective. This study aims to use CiteSpace to conduct a bibliometric analysis of the neuroimaging research on aphasia to explore the hotspots of such research and evaluate future research trends.

The application of neuroimaging to aphasia has more than 40 years of history (Lee et al., 2006), but the development process, hotspots, and trends of the future have not yet been systematic, which causes the research in this field to be characterized by fragmentation and a lack of systematic and integrative research. As a result, the hotspots and advantages of the direction are not prominent enough, which leads to a lack of researchers who understand the research status and theoretical boundaries of this field. Therefore, it is necessary to systematically identify current research hotspots, cutting-edge trends and deficiencies and then provide a reference for subsequent research.

Nevertheless, the literature research methods mostly adopt traditional literature induction, which has a good effect in literature in-depth mining, but there is a certain subjectivity. Bibliometrics is a discipline that analyzes and summarizes the research progress of the subject through mathematical and statistical methods, which can overcome the subjectivity of literature induction to a certain extent and transform a large amount of complex and disorderly literature information into a structured and orderly knowledge system to thus reveal the development law of scientific knowledge (Alajmi and Alhaji, 2018).

Therefore, to deeply understand the neuroimaging research direction of aphasia and provide more targeted guidance for later related research, we used CiteSpace and VOSviewer to conduct bibliometric research on this topic to explore the hotspots of this research and evaluate future research trends.

## Materials and methods

### Data collection

The data for this study were retrieved and extracted from Web of Science (WOS), and were downloaded within one day on October 14, 2021. The WOS database, which is constantly updated, has strict control over the published literature; therefore, it is often used in bibliometric research (Zhang et al., 2020). The data retrieval strategy was set to TS = (“aphasia”) AND TS = (“neuroimaging” OR “MRI” OR “magnetic resonance imaging” OR “fMRI” OR “functional magnetic resonance imaging” OR “sMRI” OR “structural magnetic resonance imaging” OR “DTI” OR “diffusion tensor imaging” OR “3D-T1” OR “EEG” OR “electroencephalogram” OR “ERP” OR “event related potential”). All articles that conformed to the retrieval strategy mentioned above since WOS database construction were included, while editorial material, letters and meeting abstracts were excluded.

### Data analysis and visualization

The different formats of the download file were imported into the CiteSpace and VOSviewer for analysis. CiteSpace is a document research visualization software developed by Professor Chaomei Chen. CiteSpace is used for the bibliometric analysis. The visualization result is mainly composed of nodes and links. The different nodes in the map represent elements such as disciplines and references, whose size indicates the frequency of occurrence or citation, and whose colors represent different publication years. The links between nodes represent co-operation, co-occurrence or co-citation relationships. Usually, a node with a high centrality is considered a key part of a certain subject area (Liu et al., 2015; Mulet-Forteza et al., 2019). In addition, VOSviewer was used to visually analyze the journals involved in the included articles. Besides, the number of publications per year and the overall trend were determined by Microsoft Office Excel 2019 software.

### Procedures for analysis

In this study, the parameters of CiteSpace were set as follows: time slicing (from 2004 to 2021), years per slice (1), term source (all selection), node type (choose one at a time), selection criteria (Different TOP N were set for different node types. For country, institution, cited-journals and disciplines, the TOP N we set was 50. While for keywords and cited-references, we selected top 100 of most cited), pruning (no pruning), and visualization (cluster view-static, show merged network).

In the network graph, different nodes represented various elements. Size of nodes indicates the frequency of occurrence or citation, and the connection lines between the nodes reflect the relationship between the co-operation or co-citation. Besides, the different colors within the nodes represent different times. In the analysis of clustering, the silhouette value is used to evaluate the rationality of clusters. The cluster is efficient and convincing while the silhouette value is over 0.7 (Zhong et al., 2021). In addition, centrality represents the role of a node in the knowledge network and indicates the role of the node to other nodes. Nodes with greater centrality are considered as the key nodes in the network (Zhou et al., 2021).

## Results

**FIGURE 1:**
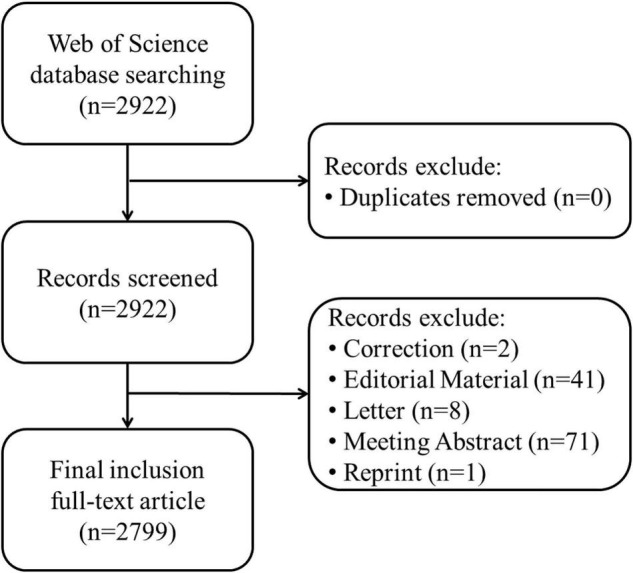
Flowchart of including and excluding publications.

**FIGURE 2:**
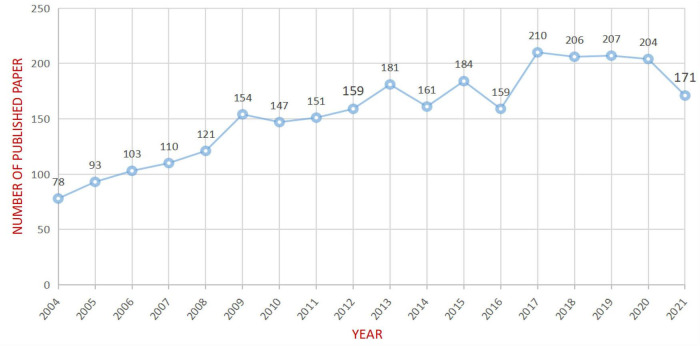
Annual trend chart of publications.

**FIGURE 3:**
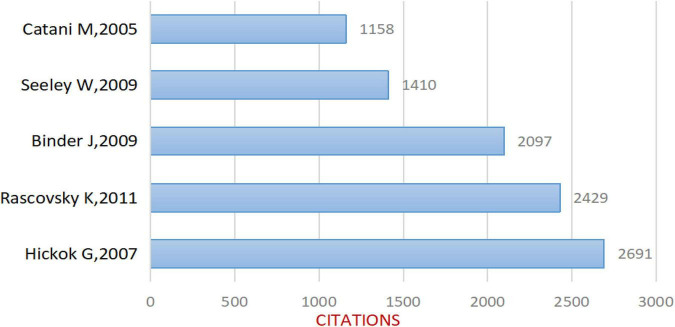
Top 5 cited articles included in the study.

After removing non-compliant types of publications, a total of 2799 articles were included in this study (publications before October 14, 2021 were included), and the time span was from 2004 to 2021 (Figure 1). As shown in Figure 2, the number of articles published has increased from 78 in 2004 to 210 in 2017. The overall trend is increasing but with some fluctuations. From 2017 to 2020, the number of articles published annually remained at more than 200. Figure 3 shows the top 5 cited documents in this study. Among them, Hickok and Poeppel (2007) “The cortical organization of speech processing” published in 2007 has the highest number of citations, which reached 2691 times.

### Distribution of countries/regions and institutions

According to the visual analysis of countries and institutions through CiteSpace, it was found that the 2,799 articles included in the study were published by 1,208 institutions in 76 countries/regions. As shown in Table 1, the United States of America (1078 articles, 38.5%), England (314, 11.2%), Germany (273 articles, 9.8%), Italy (251, 9.0%), Japan (201, 7.2%), the People’s Republic of China (176, 6.3%), France (167, 6.0%), Canada (140, 5.0%), Australia (128, 4.6%), and Spain (102, 3.7%) are the top 10 countries/regions in terms of the number of articles published. The top 5 countries for centrality are the United States of America (0.47), Germany (0.17), France (0.15), Switzerland (0.13), and Australia (0.12). It can be suggested that the United States is a major country in the study of the neuroimaging of aphasia, and it plays a role in communication in this field. However, the low centrality in other countries indicates that the degree of co-operation between countries needs to be further improved.

**TABLE 1:** Top 10 countries and institutes publishing research on neuroimaging research of aphasia.

Country ranking				Institutional ranking			
	
Rank	country	Publications	Centrality	Rank	Institution	Publications	Centrality
1	United States of America	1078	0.47	1	Univ Calif San Francisco	120	0.12
2	England	314	0.11	2	UCL	110	0.21
3	Germany	273	0.17	3	Mayo Clin	107	0.04
4	Italy	251	0.02	4	Johns Hopkins Univ	100	0.07
5	Japan	201	0.01	5	Univ Penn	90	0.07
6	People’s Republic of China	176	0.07	6	Northwestern Univ	64	0.06
7	France	167	0.15	7	Univ Manchester	57	0.05
8	Canada	140	0.08	8	Harvard Univ	48	0.07
9	Australia	128	0.12	9	Boston Univ	42	0.02
10	Spain	102	0.02	10	Univ Queensland	39	0.05
				10	Univ Vita Salute San Raffaele	39	0.06

*Univ Calif San Francisco, University of California San Francisco; UCL, University College London; Mayo Clin, Mayo Clinic; Johns Hopkins Univ, Johns Hopkins University; Univ Penn, University of Pennsylvania; Northwestern Univ, Northwestern University; Univ Manchester, University of Manchester; Harvard Univ, Harvard University; Boston Univ, Boston University; Univ Queensland, University of Queensland; Univ Vita Salute San Raffaele, Vita Salute San Raffaele University.*

In terms of publishing institutions, the research institution that published the most papers was the University of California San Francisco (120 papers), followed by University College London (110 papers), Mayo Clinic (107 papers), Johns Hopkins University (100 papers), University of Pennsylvania (90 articles), Northwestern University (64 articles), University of Manchester (57 articles), Harvard University (48 articles), Boston University (42 articles), University of Queensland (39 articles), and Vita Salute San Raffaele University (39 articles). The top 10 research institutions with the above publications are from the United States of America (7), England (2), Australia (1) and Italy (1). The top 5 institutions for centrality are UCL (0.21), University of California San Francisco (0.12), Johns Hopkins University (0.07), University of Pennsylvania (0.07), Harvard University (0.07) and University of Toronto (0.07). The results show that American research institutions have carried out continuous and in-depth research in the field of aphasia neuroimaging and have obtained rich results. At the same time, there are corresponding co-operative relations between research institutions according to the results of the centrality analysis, especially UCL and University of California San Francisco.

### Analysis of journals and cited-journals

**FIGURE 4:**
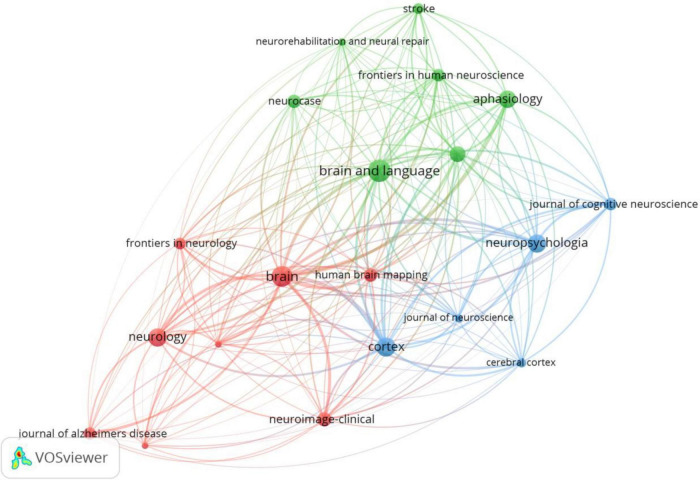
Journals network map of neuroimaging research on aphasia. Nodes represent journals and larger nodes represent more publications. Clusters are represented by different colors, and links represent co-citation relationships between journals.

We used VOSviewer to analyze the journals and found that 2799 articles were published in 486 academic journals. The analysis results suggest that Brain and Language (120) has the highest number of posts, followed by Brain (106), Cortex (91), Neuropsychologia (87), and Neurology (84) (Figure 4 and Table 2), and the journal with the highest impact factor is Brain (13.501). An analysis of cited-journals by Citespace shows that the top 5 cited journals are Brain, Neurology, Neuroimage, Annals of Neurology, and Brain and Language (Table 2), and the journal with the highest impact factor is also Brain (13.501). Through the analysis of the number of articles, the number of citations, and the impact factors, Brain has been identified as a core journal in the field of the neuroimaging research in aphasia, and its articles can reflect the current status and frontiers of the research field.

**TABLE 2:** Top 5 journals and cited-journals of neuroimaging research on aphasia.

Rank	Journal	Count	IF	JCR	Cited-Journal	Count	IF	Centrality
1	Brain and Language	120	2.381	Q4	Brain	2054	13.501	0.08
2	Brain	106	13.501	Q1	Neurology	1911	9.910	0.19
3	Cortex	91	4.027	Q2	Neuroimage	1660	6.556	0.05
4	Neuropsychologia	87	3.139	Q3	Annals of Neurology	1420	10.422	0.06
5	Neurology	84	9.910	Q1	Brain and Language	1402	2.381	0.04

*IF, Impact Factor. JCR, Journal Citation Reports.*

### Disciplines and topics involved in the neuroimaging on aphasia

**FIGURE 5:**
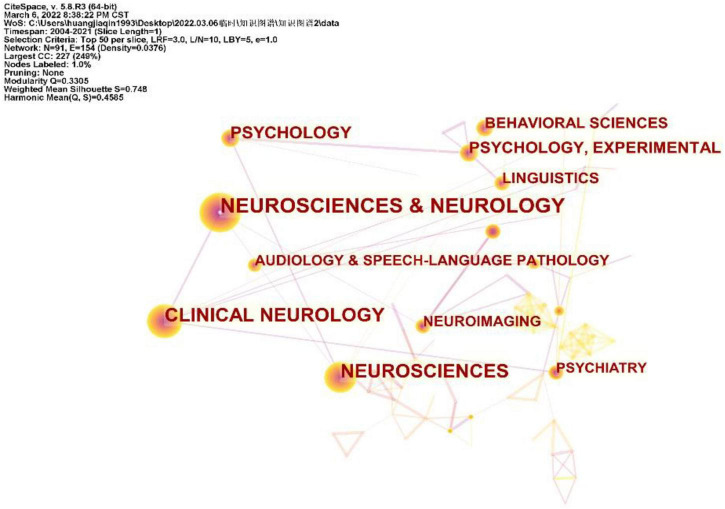
Disciplines involved in neuroimaging on aphasia, shown as a network of subject categories.

Each article indexed by WOS is assigned one or more subject categories. Figure 5 shows a network of subject categories. The most common category was Neurosciences and Neurology, which has the largest circle, followed by Clinical Neurology and Neurosciences. The presence of communication and other cognitive impairments may affect the social engagement, mental health and quality of life of patients with aphasia. Although Psychology, Behavioral Sciences and Psychiatry are much smaller, they are marked for reference.

### Analysis of keywords

**FIGURE 6:**
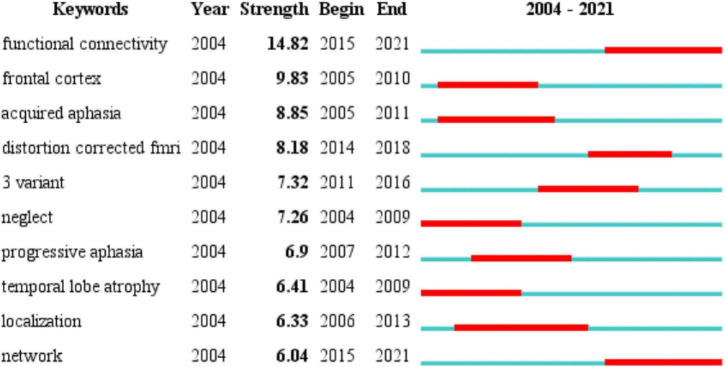
Time trends of burst keywords included in the 2799 articles. The top 10 keywords with the strongest citation bursts. Blue line represents the base timeline, and red part indicates the burst duration of each keyword.

The changes in the frequency of occurrence or the citations of keywords in a certain period can often be used as an important indicator to evaluate cutting-edge topics and trends in the field (Zheng and Wang, 2019). In addition, identifying burst keywords among all the keywords may help to predict new frontier topics or research trends in the future. Thus, to understand the development of the neuroimaging research on aphasia in a more comprehensive manner, we used CiteSpace to perform a burst keyword analysis. The top 10 keywords with the strongest burst strength from 2004 to 2021 are shown in Figure 6. The keyword with the strongest citation strength was functional connectivity (14.82), and this trend lasted for 6 consecutive years (2015−2021), which is also the latest burst keyword. Another recent burst keyword is network. The above two keywords suggest that the functional connectivity of brain networks is a research hotspot in the field. The functional connectivity of brain networks is an important basis for functioning, and neuroimaging technology has obvious advantages in observing the functional connectivity of brain networks.

### Analysis of cited-references

CiteSpace divides the co-citation network into a number of clusters of co-cited references such that citations in the same cluster are tightly connected but loosely connected in different clusters. Table 3 lists seven major clusters by their size, that is, the number of members in each cluster is greater than 100. Clusters with fewer members tend to be less representative than clusters with more members because small clusters are formed by being cited in a smaller number of publications. The quality of a cluster is also reflected in terms of its silhouette score, which is an indicator of its homogeneity or consistency. The silhouette values of homogenous clusters tend to be close to 1 (Wu et al., 2021; Zhong et al., 2021). All clusters in Table 3 are highly homogeneous. Each cluster is labeled by noun phrases from the titles of the citing articles in the cluster. Labels chosen by the log-likelihood ratio test method (LLR) are used in the subsequent discussion. The average year of publication of a cluster indicates its recentness. For example, Cluster #0 (PSA) has an average year of 2014, which is also the largest cluster. The most recently formed cluster, Cluster #2 (progressive supranuclear palsy), has an average year of 2017. Thus, we give special attention to the top five clusters (Cluster#0, Cluster#1, Cluster#3, and Cluster#4) in terms of the number of members to identify the hotspots in aphasia neuroimaging research and to the clusters (Cluster#2) with high recentness to determine the emerging trends in this field. This visualization of the network also shows high burst terms in the titles and abstracts of the references in the main clusters. For instance, the term PSA is a burst term associated with Cluster #0, which is labeled by a selection mechanism, the LLR.

**TABLE 3:** Major clusters of co-cited references.

Cluster ID	Size	Silhouette	Mean (Year)	Label (LLR)
0	237	0.849	2014	Post-stroke aphasia
1	160	0.863	2002	fMRI study
2	154	0.878	2017	Progressive supranuclear palsy
3	152	0.911	2011	Frontotemporal dementia
4	135	0.873	2006	Aphasia recovery
5	134	0.891	2005	Frontotemporal lobar degeneration
6	110	0.944	2002	Neural region

**FIGURE 7:**
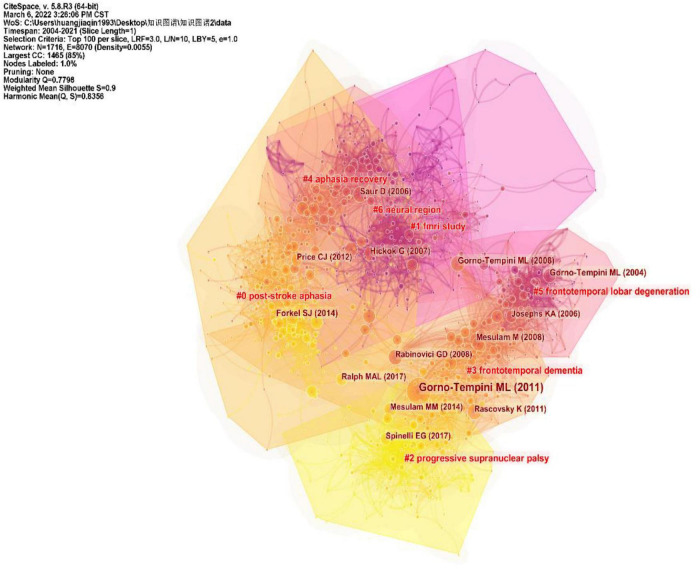
Trajectories of relevant research shown in a hybrid network of co-cited references and burst terms from titles and abstracts.

Figure 7 shows a visual network of co-citations and burst terms for the neuroimaging studies of aphasia. Each cluster contains information about cited references and citing articles. To further show the research focus and new trends in this field, this paper lists the key components of the major Clusters #0, #1, #2, #3, and #4 (Tables 4–8). The five most cited articles and the five most cited references in Clusters #0, #1, #3, and #4 are highlighted. Since Cluster #2 is more representative of the latest trends, ten citing articles with the most references in a cluster are selected, whereas ten cited references with the most citations are highlighted in Cluster #2.

**TABLE 4:** Cited references and citing articles of Cluster #0 on post-stroke aphasia.

Cluster #0 post-stroke aphasia			

Cited References		Citing Articles	
	
Cites	Author (Year) Journal, Volume, Page	Coverage%	Author (Year) Title
50	Forkel SJ (2014) Brain, 137, 2027	39	Stockert, Anika (2016) Insights into early language recovery: from basic principles to practical applications
43	Ralph MAL (2017) Nat Rev Neurosci, 18, 42	29	Kiran, Swathi (2019) Neuroplasticity of language networks in aphasia: advances, updates, and future challenges
40	Saur D (2008) P Natl Acad Sci USA, 105, 18035	28	Hartwigsen, Gesa (2019) Neuroimaging of stroke recovery from aphasia−insights into plasticity of the human language network
38	Ivanova MV (2016) Cortex, 85, 165	22	Wilson, Stephen M (2019) Multivariate approaches to understanding aphasia and its neural substrates
35	Kummerer D (2013) Brain, 136, 619	22	Roelofs, Ardi (2014) A dorsal-pathway account of aphasic language production: the weaver plus plus/arc model

**TABLE 5:** Cited references and citing articles of Cluster #1 on functional magnetic resonance imaging (fMRI) study.

Cluster #1 fMRI study			

Cited References		Citing Articles	
	
Cites	Author (Year) Journal, Volume, Page	Coverage%	Author (Year) Title
55	Hickok G (2007) Nat Rev Neurosci, 8, 393	47	Demonet, JF (2005) Renewal of the neurophysiology of language: functional neuroimaging
22	Hickok G (2004) Cognition, 92, 67	25	DeLeon, Jessica (2007) Neural regions essential for distinct cognitive processes underlying picture naming
18	Indefrey P (2004) Cognition, 92, 101	22	Duffau, H (2005) New insights into the anatomo-functional connectivity of the semantic system: a study using cortico-subcortical electrostimulations
16	Dronkers NF (2004) Cognition, 92, 145	22	Amici, Serena (2007) Performance in specific language tasks correlates with regional volume changes in progressive aphasia
16	Bookheimer S (2002) Annu Rev Neurosci, 136, 619	20	Skipper, JI (2005) Listening to talking faces: motor cortical activation during speech perception

**TABLE 6:** Cited references and citing articles of Cluster #2 on progressive supranuclear palsy.

Cluster #2 progressive supranuclear palsy			

Cited References		Citing Articles	
	
Cites	Author (Year) Journal, Volume, Page	Coverage%	Author (Year) Title
45	Spinelli EG (2017) Ann Neurol, 81, 430	46	Peet, Bradley T (2021) Neuroimaging in frontotemporal dementia: heterogeneity and relationships with underlying neuropathology
29	Kumfor F (2016) Brain, 139, 986	40	Dev, Sheena I (2021) Neuroimaging in frontotemporal lobar degeneration: research and clinical utility
24	Mandelli ML (2016) Brain, 139, 2778	24	Whitwell, Jennifer L (2019) Ftd spectrum: neuroimaging across the ftd spectrum
22	Botha H (2015) Cortex, 69, 220	21	Montembeault, Maxime (2018) Clinical, anatomical, and pathological features in the three variants of primary progressive aphasia: a review
21	Lowe VJ (2016) Acta Neuropathol Com, 4, 0	16	Utianski, Rene L (2018) Tau-pet imaging with [18f]av-1451 in primary progressive apraxia of speech
19	Ossenkoppele R (2016) Brain, 139, 1551	16	Schaeverbeke, Jolien (2018) Single-word comprehension deficits in the non-fluent variant of primary progressive aphasia
15	Collins JA (2017) Brain, 140, 457	14	Schaeverbeke, Jolien (2018) Distinct [f-18]thk5351 binding patterns in primary progressive aphasia variants
15	Josephs KA (2018) Ann Neurol, 83, 599	14	Tee, Boon Lead (2019) Primary progressive aphasia: a model for neurodegenerative disease
15	Santos-Santos MA (2016) Jama Neurol, 73, 733	13	Tsai, Richard M (2019) F-18-flortaucipir (av-1451) tau pet in frontotemporal dementia syndromes
14	Migliaccio R (2016) Plos One, 11, 0	13	Lukic, Sladjana (2021) Dissociating nouns and verbs in temporal and perisylvian networks: evidence from neurodegenerative diseases

**TABLE 7:** Cited references and citing articles of Cluster #3 on frontotemporal dementia.

Cluster #3 frontotemporal dementia			

Cited References		Citing Articles	
	
Cites	Author (Year) Journal, Volume, Page	Coverage%	Author (Year) Title
172	Gorno-Tempini ML (2011) Neurology, 76, 1006	33	Rohrer, Jonathan D (2012) Structural brain imaging in frontotemporal dementia
57	Gorno-Tempini ML (2008) Neurology, 71, 1227	29	Rohrer, J D (2011) Primary progressive aphasia-defining genetic and pathological subtypes
47	Rabinovici GD (2008) Ann Neurol, 64, 388	28	Agosta, Federica (2012) Neuroimaging findings in frontotemporal lobar degeneration spectrum of disorders
46	Rascovsky K (2011) Brain, 134, 2456	26	Whitwell, Jennifer L (2012) Recent advances in the imaging of frontotemporal dementia
44	Mesulam MM (2014) Brain, 137, 1176	25	Harciarek, Michal (2011) Primary progressive aphasias and their contribution to the contemporary knowledge about the brain-language relationship

**TABLE 8:** Cited references and citing articles of Cluster #4 on aphasia recovery.

Cluster #4 aphasia recovery			

Cited References		Citing Articles	
	
Cites	Author (Year) Journal, Volume, Page	Coverage%	Author (Year) Title
60	Saur D (2006) Brain, 129, 1371	34	Berthier, Marcelo L (2011) Recovery from post-stroke aphasia: lessons from brain imaging and implications for rehabilitation and biological treatments
43	Price CJ (2012) Neuroimage, 62, 816	28	Cappa, Stefano F (2011) The neural basis of aphasia rehabilitation: evidence from neuroimaging and neurostimulation
41	Baker JM (2010) Stroke, 41, 1229	24	Fridriksson, Julius (2006) Neural recruitment associated with anomia treatment in aphasia
39	Naeser MA (2005) Brain Lang, 93, 95	23	Meinzer, Marcus (2011) Recent developments in functional and structural imaging of aphasia recovery after stroke
35	Price CJ (2005) Curr Opin Neurol, 18, 429	23	Hillis, Argye E (2007) Aphasia−progress in the last quarter of a century

The core literature of Cluster #0 represents an important milestone related to PSA. Notably, Forkel et al’s. (2014) article “Anatomical predictors of aphasia recovery: a tractography study of bilateral perisylvian language networks” occupies an important place in this cluster. Their study was the first to prospectively investigate the anatomic predictors of language recovery by using diffusion tensor imaging tractography. They observed that the volume of the long segment of the right hemispheric (contralateral) arcuate tract was an important predictor of speech recovery after stroke and found that the volume of the long segment of the arcuate fasciculus in the right hemisphere was an important predictor for disease recovery.

Cluster #1, fMRI study, with a mean year of 2002, mainly contains literature that explores language processing models in conjunction with neuroimaging, which is the basis for the future neuroimaging of aphasia. Among them, Hickok and Poeppel (2007) article on “The cortical organization of speech processing” has played an important role in the cluster, which is also the most cited article in the literature included in this study (Figure 3). Hickok G’s team mainly proposed a dual-stream model of speech processing, which is a milestone work in the neuroimaging research of aphasia.

Cluster #2 is the most recently formed cluster. We presented the 10 most cited references in this cluster and 10 citing articles (Table 6). Most of the articles in this cluster aimed to explore the objective differences in brain structure and function in different clinical types of PPA combined with neuroimaging techniques. Notably, many of the studies in the cluster integrated PET/CT technology into the research of disease characteristics, which has important milestone significance.

Primary progressive aphasia is the main clinical type of aphasia, and its clinical typing diagnosis has always been a research bottleneck to be overcome, which is shown in Cluster #3. The most cited article, Gorno-Tempini et al’s. (2011) “Classification of primary progressive aphasia and its variants”, explored the differentiation of PPA variants from clinical, imaging-supported, or definite pathologic diagnosis. Their work emphasized the importance of biomarkers (e.g., molecular PET imaging or CSF markers) in the diagnosis of PPA variants and provided a normative framework for the classification of PPA’s major variants to promote consistency in clinical diagnosis.

Cluster #4, aphasia recovery, is related to the recovery mechanism of aphasia, which is also an important area of neuroimaging in aphasia research. Saur et al. (2006), as the most critical component of this cluster, revealed the dynamics of reorganization in the language system of aphasia in different periods (acute phase, subacute phase and chronic phase) combined with fMRI. In addition, Naeser et al. (2005); Baker et al. (2010) conducted a clinical study on the efficacy of two main treatments (anodal transcranial direct current stimulation and repetitive transcranial magnetic stimulation) based on the characteristics of the brain region with nerve injury in aphasia, since the two treatment methods have the advantage of precise positioning.

## Discussion

### General condition of the neuroimaging study of aphasia

Based on the bibliometric research methods that used CiteSpace, our study explored the characteristics of aphasia neuroimaging from the literature related to the neuroimaging research on aphasia published from 2004 to 2021. From 2004 to 2017, the number of articles on aphasia neuroimaging research showed an overall upward trend, while the volume of articles published each year remained at a relatively high level from 2017 to 2020, which indicates that the application of neuroimaging technology to the study of aphasia has attracted increasing attention from researchers, and this topic may continue to be a research hotspot for scholars in the future (Figure 2).

Judging from the national sources of the literature (Table 1), only China (ranked 6th) has entered the top 10 as a developing country, and the rest are developed countries. Notably, the top 10 publishing agencies are all developed countries. Therefore, developing countries need to strengthen scientific research investment, promote research co-operation, and increase attention to related fields. Another finding of our research was that Brain (IF = 13.501) is the journal with the highest ranking in both publications and citations, while Neurology (IF = 9.910) is the most centrally cited journal, which indicates that these two journals have an important position, and their articles are the basis of the research direction (Figure 4 and Table 2). In addition, the disciplines and topics of this study involved Psychology, Behavioral Sciences and Psychiatry. Patients with PSA have other non-verbal cognitive dysfunctions and affective disturbances (El Hachioui et al., 2014; Mitchell et al., 2017), which may account for the disciplinary classification of neuroimaging studies in aphasia.

From the time trends of the burst keywords included in the 2799 articles, we can see that the recent burst keywords are functional connectivity and network. These two keywords suggest the main use of neuroimaging in aphasia, which is to objectively present the functional connections of the brain network to provide scientific evidence for the changes in brain networks in language processing and the occurrence and recovery of aphasia.

With the advantage of technology, what is the application of neuroimaging in aphasia? On the one hand, CiteSpace and VOSviewer provide the tools for our research by showing how a scientific field evolves over time through a synthetic literature network. On the other hand, if two references are frequently cited together, then they are intrinsically related. There is a double relationship between cited documents. It has been shown that network information formed in this way has the function of revealing a potential research focus. Therefore, we identify hot issues in the neuroimaging studies of aphasia based on the results of the cited literature network produced by CiteSpace.

### The basis of language processing−discovery of the dual-stream model

Language processing has been the focus of medical attention and research (Henderson et al., 2017; Thompson, 2019), and explaining the internal mechanism of language phenomena will help us to understand the brain damage mechanism of aphasia. In recent years, the development of imaging technology has provided support for the study of the brain mechanism of language function, and research on language processing has made great achievements. However, a large number of studies lack systematic sorting, which results in the emergence of many language models, such as the “Wernicke-Lichtheim-Geschwind” model (Weems and Reggia, 2006), DIVA model (Terband et al., 2020), and the dual-stream model (Fridriksson et al., 2018). Which model has been influencing aphasia research for a long time?

In our study, Cluster #1, fMRI study, as the second largest cluster, was also a cluster with an earlier average publication year, which suggests that the literature in this cluster may be an important basis for the neuroimaging studies of aphasia. The most cited article in Cluster #1, Hickok and Poeppel (2007) “The cortical organization of speech processing”, summarized the speech processing of a dual-stream model and proved that the dual-stream model plays a prominent role among the above models from the perspective of bibliometrics. The dual-stream model was developed in Hickok and Poeppel (2004) earlier paper (the second-most cited article in Cluster #1), in which Hickok G described the origins of the dual-stream model. Drawing on the cortical organization of the visual processing streams, they developed a new model of language functional anatomy by thinking about two aspects of language processing (sensory-conceptual and sensory-motor) and summarized the recent experimental evidence related to this model. He then argued that this cortical processing system is split into two main processing streams, namely, a ventral stream, which maps sound to meaning, and a dorsal stream, which maps sound to sound-based representations. However, they proposed a language model so broad that many details (from sound to language production) are not taken into account. Meanwhile, the role of the frontal and subcortical systems in language processing was not included in this language model. As a result, the early dual-stream model was not precise and specific and failed to completely cover language processing.

Then, in Hickok and Poeppel (2007) team reintegrated previous research and outlined the core components and assumptions of the dual-stream model to address the above situation. The revised model involves a broader part of the cerebral cortex, including the frontal, temporal and parietal lobes. The ventral stream includes structures in the superior and middle portions of the temporal lobe that process speech signals for comprehension. The dorsal stream involves structures in the posterior frontal lobe and the posterior dorsal-most aspect of the temporal lobe and parietal operculum that map acoustic speech signals into articulatory representations in the frontal lobe. This work not only refined the components of the model in scope but also integrated a wide range of experimental studies to clarify the details of language processing. In general, their dual-stream model of language processing encouraged researchers to explore the details of the organization and computational operations within the model.

In Saur et al. (2008) article (one of the highly cited references in Cluster #0) showed that they initially constructed the central brain area distribution of the dual-stream model by using t-fMRI and DTI, and they found that the dorsal stream is the superior temporal cortex and the frontal motor cortex, which are connected by the arcuate fasciculus and the upper longitudinal beam, while the ventral stream is the middle temporal gyrus and the ventrolateral prefrontal cortex, which are connected by the outermost capsule. On the basis of Saur D’s research, Faria et al. (2012) used the JHU brain area template as the basis for brain area division and constructed a more specific and reliable dual-stream model area distribution to resolve the brain area distribution dispute (Fridriksson et al., 2016). Accordingly, their research further enriched the anatomical basis of the dual-stream model of language. Even though the same results were continually repeated, this type of study was still significant as it demonstrated the remarkable consistency of language function anatomy across many studies. With the in-depth application of neuroimaging technology in the exploration of language processing, language models are constantly being improved and provide a key framework for future studies on the mechanisms of aphasia injury and recovery.

However, language processing includes a series of subtasks, such as auditory processing, articulation, visual processing, and semantics (Price, 2012). The role of cognitive models in language is also being explored (Ralph et al., 2017). As a highly cited article in Cluster #1, Indefrey and Levelt (2004) introduced a comprehensive meta-analysis of neuroimaging studies on word generation (82 experiments). Considering the spatial distribution of activations, the time course of activations in picture naming and the chronometric data, they found a flow pattern of activation during word formation. Research into the anatomical basis of specific language functions is also a focus of study. For example, Dronkers et al. (2004) analyzed brain damage related to language comprehension in PSA patients and found that the middle temporal gyrus may be more important for language comprehension. Although similar studies have been carried out, the overall process of language processing remains to be further explored due to its complexity. Future research will aim to clarify the precise spatial distribution of different language functions, but this is a great challenge due to the spatial overlap of activated brain regions, the sequence of time series and functional interactions.

### The main research objects in the neuroimaging studies of aphasia-post-stroke aphasia and primary progressive aphasia

The causes of aphasia are diverse, which leads to different causes of aphasia with a specific pathogenesis. Therefore, it is necessary to clarify the main clinical types of aphasia in this study to explore the hotspots and trends of the neuroimaging research on aphasia more accurately. According to the results of the cited-references analysis, we mainly listed the clusters that were greater than 100 (Table 3) and conducted an in-depth analysis of the top five clusters. Researchers mainly focused on two clinical types of aphasia, specifically, PSA (Clusters #0 and #4) and PPA (Clusters #2 and #3), in the neuroimaging studies of aphasia. Next, our discussion focuses on the hotspots concerning PSA and PPA.

### Exploring the mechanism of injury and recovery in post-stroke aphasia with neuroimaging techniques

Stroke is a common main cause of aphasia (Stefaniak et al., 2020), and approximately one-third of stroke patients will experience aphasia (Engelter et al., 2006; Flowers et al., 2016). A number of studies have found that the different manifestations of PSA are closely related to changes in the function and structure of related brain regions. Therefore, it is of great significance to clarify the brain injury mechanism of different symptoms missing in PSA patients for more accurate localization diagnosis and clinical treatment. In Cluster #0, we found two interesting studies, Ivanova et al. (2016) “Diffusion-tensor imaging of major white matter tracts and their role in language processing in aphasia” and Kummerer et al. (2013) “Damage to ventral and dorsal language pathways in acute aphasia”. As the most representative articles on the injury mechanism of PSA in the cluster, these two articles used the language dual-stream model as the template. Kummerer et al. (2013) tested the association of acute repetition and comprehension disorders with dorsal or ventral flow lesions in 100 patients with PSA. Their results from acute stroke patients supported the idea that language is organized along two segregated dorsal-ventral streams, which provides neuroimaging evidence for the application of a dual-stream model in PSA. Meanwhile, Kummerer et al. (2013) for the first time, proved that the auditory understanding function was related to the interaction between the temporal and prefrontal brain regions *via* the ventral extreme capsule pathway. Kummerer et al’s. (2013) work not only validated the importance of a dual-stream model for the injury mechanism of PSA but also provided a research paradigm to further explore the anatomical basis of language processing based on a dual-stream model. In addition, subcortical white matter may also affect the production of language (Crosson et al., 2003; Bonilha et al., 2014). Ivanova et al. (2016) undertook the first broad examination of major white matter tracts in PSA. They emphasized the importance of fiber pathways in supporting different language functions and the necessity of examining individual small tract segments to accurately explore the role of fiber pathways. Ivanova MV’s study was the first to explore the mechanism of white matter injury in PSA based on the dual-stream model and brought researchers’ attention to fiber connections in the pathogenesis of PSA. Accordingly, the work of Kummerer D and Ivanova MV suggests that it is a hotspot and a future trend to explore the damaging mechanism of PSA in combination with the language function and anatomy by using the dual-stream model as a template.

Cluster #4, aphasia recovery, with the average publication year of 2006, indicated that neuroimaging techniques had been applied in the recovery of aphasia for a long time. By investigating the literature in Cluster #4, we found that the neuroimaging studies on aphasia recovery mainly focused on exploring the recovery mechanism of PSA and the efficacy mechanism of effective PSA treatments. The severity of aphasia is reflected in the interaction of many factors that lead to a decline in the quality of life, and barriers to language communication often cause changes in mood and social skills. Researchers have investigated the quality of life of residents who require long-term care for 60 diseases and 15 conditions and demonstrated that aphasia has the greatest negative correlation with quality of life (Lam and Wodchis, 2010). Regarding PSA, its occurrence affects the development of stroke disease, which leads to increased mortality, functional recovery obstacles and increased medical expenditure (Dickey et al., 2010; Ellis et al., 2012). Therefore, a comprehensive understanding of aphasia recovery has considerable clinical and social significance. The results of the cited-references clustering contribute to understanding the rehabilitation process of PSA from the perspective of influencing factors and recovery mechanisms.

Various factors interfere with the rehabilitation of aphasia, including disease-related factors, patient factors and treatment-related factors (Watila and Balarabe, 2015). Notably, the most cited literature in Cluster #0, Forkel SJ’s “Anatomical predictors of aphasia recovery: a tractography study of bilateral perisylvian language networks,” explored the value of measurements of different segments in the arcuate fasciculus in predicting speech recovery 6 months later in patients with left hemisphere stroke. Their study demonstrated that the right hemisphere language network plays an important role in aphasia recovery after a stroke in the left hemisphere. Forkel SJ’s work is an important milestone in the research on the mechanisms of aphasia recovery. On the one hand, their research method provides a reference and guidance for the further exploration of more accurate anatomic predictors in PSA by neuroimaging techniques in the future. On the other hand, their results indicate the role of the right hemisphere in PSA recovery, which may be related to functional compensation.

Neuroimaging techniques, especially multimodal nuclear magnetic imaging, also play an important role in the study of PSA recovery mechanisms. Saur et al. (2006), the most cited references in Cluster #4, clarified the dynamics of reorganization in the language system of PSA patients by repeated fMRI examinations at different time periods as follows: the activation of non-infarcted left-hemispheric language structures in the acute period decreased significantly; in the subacute phase, the bilateral language network was significantly activated, especially in the right Broca homolog; and in the chronic phase, the activation peak moved back to the left hemisphere language region, and the normalization of activation was observed. On the one hand, their work emphasized the role of the left and right cerebral hemispheres in the recovery of language function and provided a framework for language recovery models. Since then, numerous researchers have carried out a series of studies on the neural network recovery mechanism of PSA through neuroimaging technology. The comprehensive research results indicate that the recovery of PSA may be related to the following three types of neuroplastic changes: the (a) functional recovery and reconstruction of language processing in the injured part of the left cerebral hemisphere and its surrounding area (Meinzer et al., 2011; Torres et al., 2013; Anglade et al., 2014); (b) activation and reorganization of the language mirror area in the right cerebral hemisphere (Thiel and Zumbansen, 2016); and (c) activation of the non-dominant hemisphere inhibits the recovery of language function (Breier et al., 2009; Martin et al., 2009).

On the other hand, Saur D’s findings had implications for the clinical treatment of PSA in different phases. Notably, the study that has the highest citation coverage of 34% in Cluster #4 is an article by Berthier et al. (2011), which suggested different treatment principles of PSA language rehabilitation in PSA. They proposed that during acute stroke, reperfusion mechanisms are responsible for restoring language function, and it is necessary to restart functional activity as quickly as possible to activate the relevant brain regions. In chronic PSA, most neuroimaging studies have explored the role of speech language therapy (SLT) and repeated transcranial magnetic stimulation (rTMS) in triggering functional plasticity. Our study found that two highly cited articles (Naeser et al., 2005; Baker et al., 2010) in Cluster #4 (Baker JM’s article and Naeser MA’s article) discussed the efficacy of transcranial direct current stimulation (tDCS) and rTMS, which are two popular rehabilitation technologies, in PSA patients. tDCS and rTMS, as two clinically valid methods of non-invasive brain stimulation, have the characteristics of adjusting the stimulation dosing and stimulation position according to the size and distribution of the lesion site (Torres et al., 2013). Accordingly, current studies have discovered several activation conditions in the left and right cerebral hemispheres during PSA recovery through neuroimaging techniques, with more profound considerations for the clinical treatment of PSA based on the mechanism of recovery.

### Neuroimaging technology is expected to provide a more objective and accurate diagnosis of primary progressive aphasia

Primary progressive aphasia is another clinical type of aphasia studied in neuroimaging, according to our clustering results, whose research hotspots and trends are introduced in Clusters #2 and #3. PPA is a group of distinct disorders that collectively manifest as a relatively focal degeneration of the brain systems that control language (Marshall et al., 2018), which can be divided into the three main clinical subtypes of non-fluent/agrammatic, semantic, and logopenic variants (Jung et al., 2013). The most cited article in Cluster #3 was the 2011 International Consensus Guidelines compiled by Gorno-Tempini et al. (2011), which is also the most cited literature among these studies. Gorno-Tempini et al’s. (2011) work provided a common classification framework for PPA and its major clinical variants to promote clinical diagnostic consistency and multicenter studies. They defined the typing diagnosis of PPA from the three levels of clinical, imaging-supported and definite pathologic diagnosis and listed the work suggestions, which had been widely recognized by a large number of experts. Subsequent investigations of clinical diagnosis by this method have reported successful implementation of the guidelines. This guideline had a leading role in the clinical diagnostic studies of PPA variants, and subsequent investigations of clinical diagnosis with this method have reported a successful implementation of the guidelines (Mesulam et al., 2012; Sajjadi et al., 2012; Wicklund et al., 2014).

Based on the analysis of the references with a high citation rate in Cluster #3, logopenic progressive aphasia was given the most attention by researchers among the three types of variations. Because progressive non-fluent aphasia and semantic dementia are too simple to classify as clinical variants of PPA, the term “logopenic” was revived to label PPA patients (Weintraub et al., 1990). In Gorno-Tempini et al. (2008) explored the neuroanatomical and pathological basis of the logopenic variant (LPA) and showed that the loss of phonological loop functions may be the core mechanism of the clinical syndrome of LPA and that Alzheimer’s disease may be the most common pathological basis of the clinical syndrome of LPA. However, regarding the pathological basis of LPA, Gorno-Tempini et al. (2008) only carried out a literature review without an in-depth study. Earlier studies have found that LPA patients showed maximal atrophy in the left temporoparietal junction (Gorno-Tempini et al., 2004), which is similar to the pattern reported in AD (Karas et al., 2003). In addition, the apolipoprotein E4 genotype is highly expressed in LPA patients (Gorno-Tempini et al., 2004). Based on the above imaging, Rabinovici et al. (2008) speculated that LPA might be related to the underlying pathology of AD. They studied Aβ amyloidosis in three variants of PPA using [11C] PIB and proved that LPA is associated with Aβ amyloidosis. The results confirmed their suspicion that the underlying pathology of LPA is associated with AD and suggested that the clinical classification of PPA based on linguistic characteristics could help predict the underlying AD pathology. Additionally, highly cited in Cluster #3, Mesulam et al. (2014) reported the autopsy results of 35 patients with PPA and found that tissue diagnoses included Alzheimer’s disease and frontotemporal lobar degeneration (FTLD). FTLD included two main categories, namely, FTLD-Tau and FTLD-TDP, which is consistent with other research results (Mackenzie et al., 2010). They demonstrated that the clinical characteristics of PPA could improve the accuracy of potential pathological diagnosis by exploring the relationship between pathology and the clinical symptoms of aphasia. More significantly, their work revealed that some patients cannot be classified according to the 2011 guidelines, while others fit both subtypes, which suggests a revision of the criteria for logopenic PPA.

Accordingly, the clinical variant typing diagnosis of PPA has important research value and is also the focus of researchers’ attention. In addition to clinical features, neuroimaging techniques provide imaging support for the diagnosis of PPA variants to finally improve the accuracy of diagnosis (Botha and Josephs, 2019). Pathological diagnosis is also an important diagnostic basis for PPA variants, and its deeper value needs to be further explored. However, previous neuroimaging techniques have realized the use of PET/CT to observe Aβ amyloidosis in brain tissue (Rabinovici et al., 2008), but studies of other disease-specific proteinopathies are mostly carried out in autopsy (Mesulam et al., 2008; Gliebus et al., 2010), which limits the development of the pathological diagnosis of PPA variants. Therefore, the research trend at the time was to promote the development of neuroimaging technology and strive to realize the imaging of key disease-specific proteins on living objects.

### Emerging trends

Cluster #2 is the most recently formed cluster with the strongest recentness (Chen et al., 2012). We conduct an in-depth analysis of the top 10 most highly cited references in Cluster #2 to identify new trends in the neuroimaging studies of aphasia. The diagnosis of the clinical variant typing of PPA is still the main research content of the literature in Cluster #2. Recent studies are still trying to further clarify the clinical symptoms, imaging and pathological features of different PPA variants based on previous studies. The difference is that the latest study focuses more on exploring the relationship between the characteristics of the above three aspects, especially the role of neuroimaging in pathological diagnosis. Several researchers have explored the diagnostic value of clinical symptoms and multimodal imaging in PPA and its variants. Botha H conducted a prospective assessment of PPA and Apraxia of Speech using a multidisciplinary clinical assessment and multimodal imaging to provide a new reference for the identification and classification of diseases (Botha et al., 2015). In addition, Kumfor et al. (2016) found the degeneration of the right anterior temporal and orbitofrontal cortices by analyzing the correlation between the clinical features of semantic dementia and the atrophy of brain regions, which emphasizes the role of these regions in social cognition and behavior.

However, as PPA is a neurodegenerative disease, the concept of clinicopathological relevance is crucial in distinguishing PPA variants. Ideally, the clinical variants of PPA should accurately reflect their respective underlying pathologies (Harris and Jones, 2014). The difficulty in obtaining pathological tissue of the human brain limits the diagnostic value of the pathological examination of PPA. Therefore, Spinelli et al’s. (2017), the most cited article in Cluster #2, investigated the predictive value of an automatic classification algorithm based on MRI variables for PPA pathological diagnosis to address the above situation. They used support vector machine analysis and found that the accuracy of distinguishing between the FTLD-Tau and FTLD-TDP variants combined with white matter and gray matter volumes was up to 92.7%, which was the first study in which machine learning was applied to the pathological diagnosis of PPA. Mandelli et al. (2016) in Cluster #2 indirectly provides support for the predictive value of MRI variables in PPA pathological diagnosis. As many studies have shown, the transsynaptic spread of abnormally folded proteins through connected neuronal pathways influences specific atrophy patterns in neurodegenerative diseases (de Calignon et al., 2012; Liu et al., 2012). Thus, the disease may spread along the path most closely connected to the lesion’s central region and reach the other regions through the architecture of predetermined large-scale neuronal networks (Raj et al., 2012). By comparing the connectivity of atrophy pattern changes in patients with functional and structural speech/language networks, Mandelli ML’s article supports the hypothesis that the spread of neurodegeneration may have specific anatomical and functional brain network pathways. Similarly, Collins et al. (2017) also demonstrated that cortical atrophy in semantic variant PPA may also occur along a large-scale network path. The above studies have proven to some extent that multimodal MRI technology can reflect the distribution and function of disease-specific proteins by explaining that the brain function and structure presented by MRI technology are correlated with pathological diagnosis.

Despite the pioneering significance of exploring the predictors of pathological diagnosis based on clinical symptoms and MRI variables, the use of these indirect methods in the pathological diagnosis of PPA variants is imprecise and cannot directly reflect the distribution and function of disease-specific proteins in brain tissues. In the past, the ability to detect Aβ protein deposition using the [11C] PIB had an important impact on the determination of PPA pathologic types (Rabinovici et al., 2008), whereas the detection of other disease-specific proteins had yet to be broken through. Tau, for example, acts as a downstream promoter of Aβ protein (Jack and Holtzman, 2013) and has often been suggested to have a damaging effect on synapses (Beharry et al., 2014; Spires-Jones and Hyman, 2014). In our study, we found that one of the highly cited articles within Cluster #2, Ossenkoppele et al. (2016) “Tau PET patterns mirror clinical and neuroanatomical variability in Alzheimer’s disease,” used 18F-AV1451 (a recent PET tracer) to map the pathological distribution of the Tau protein in the brain tissues of Alzheimer’s disease patients. Consequently, the advent of a new tracer, 18F-AV1451, enables the visualization of Tau deposition in the living brain. Several studies have shown that the three different Tau subtypes of 4R tau, 3R tau and 3R + 4R tau contribute to the diagnosis of PPA variants (Josephs et al., 2006; Mesulam et al., 2014; Spinelli et al., 2017). Therefore, another highly cited article published by Josephs KA in Cluster #2 revealed that the uptake pattern of 18F-AV1451 tau-PET was different across the PPA variants and showed excellent differentiation ability among the variants (Josephs et al., 2018). Based on the work of Josephs KA, Schaeverbeke et al. (2018), evaluated the diagnostic ability of another tau PET ligand, [18F]THK5351, for PPA variants (one of the articles in the citing articles list in Cluster #2) and found that [18F]THK5351 binding was associated with the severity of language impairment.

Accordingly, the emerging trend is to focus more on defining the characteristics of PPA variants, including the clinical symptoms, imaging support and pathology, with a particular emphasis on the value of pathology. This new trend is of great significance for the accurate diagnosis of PPA variants and provides a reference for clinical modification therapy of PPA in the future. The development of PET tracer technology has provided us with a non-invasive and effective tool for observing disease-specific proteins. Future studies should further clarify the pathological diagnosis of PPA and its relationship with clinical features and MRI imaging in conjunction with evolving neuroimaging techniques.

### Limitations and prospects

Several limitations of our study should be pointed out. First, we analyzed only relevant publications in the WOS due to the software limitations, so the data may not be comprehensive. Secondly, only English articles were analyzed in this study, as articles in other languages were not considered. Finally, the existence of synonyms may lead to the overlapping of different categories of words when clustering. Nevertheless, the analysis of literatures *via* CiteSpace has the advantage of visualizing the data, and helps to explore the history, current status, and focus of research areas in greater depth than traditional reviews. Therefore, we believe that our analysis can reveal the future trends and hot spots in the neuroimaging in aphasia to a certain extent. It is worth pointing out that it is necessary to include multiple literature databases and articles in multiple languages in future research. And careful review of the included articles and in-depth analysis of the key literatures are also needed to explore the hot spots and frontiers of the neuroimaging researches in aphasia more completely.

## Conclusion

CiteSpace is a visualization software for literature analysis, with which we performed an approximate analysis of the results found in this research. Global neuroimaging research on aphasia was in a state of continued popularity from 2004 to 2020. In general, in this bibliometric study, we determined the following three hotspots of neuroimaging research on aphasia according to this bibliometric method: the dual-stream model of language processing; the mechanism of injury and recovery of PSA, and the diagnostic criteria for PPA variants. In addition, from the most recently formed cluster of cited-references clustering, we found that the visualization of disease-specific proteins of PPA variants based on new neuroimaging techniques is the focus of future research. Therefore, we demonstrated a scientometric approach to exploring the progress of collective knowledge by mining the literature on the neuroimaging studies of aphasia, and our results provide directions and prospects for neuroimaging research on aphasia in the future.

## Author contributions

JH and JC proposed this idea. YC completed the data collection. JH conducted the data analysis and drafted the manuscript. JC proposed key revisions to the manuscript. DZ and XL participated in the revision of the manuscript. All authors participated in the discussion of the research results and formulation of the plan and contributed to this article and approved the submitted version.

## Conflict of Interest

The authors declare that the research was conducted in the absence of any commercial or financial relationships that could be construed as a potential conflict of interest.

## Publisher’s Note

All claims expressed in this article are solely those of the authors and do not necessarily represent those of their affiliated organizations, or those of the publisher, the editors and the reviewers. Any product that may be evaluated in this article, or claim that may be made by its manufacturer, is not guaranteed or endorsed by the publisher.
